# Trends in mortality of the WHO-recommended diseases for palliative care in the Republic of Korea, 2014–2023

**DOI:** 10.3389/fpubh.2026.1752495

**Published:** 2026-02-11

**Authors:** Minju Kim, Kyuwoong Kim, Woorim Kim, Eun Jeong Nam, Su Yeon Kye, Jin Young Choi

**Affiliations:** 1 National Cancer Control Institute, National Cancer Center, Goyang, Republic of Korea; 2 Graduate School of Cancer Science and Policy, National Cancer Center, Goyang, Republic of Korea

**Keywords:** cause of death, mortality, palliative care, Republic of Korea, World Health Organization

## Abstract

**Background:**

The global burden of diseases requiring palliative care is increasing due to aging populations and rising non-communicable diseases. The WHO predicts an 87% increase in serious health-related suffering by 2060. In the Republic of Korea, more than half of the leading causes of death require palliative care. This study aims to analyze 10-year mortality trends and changes in places of death for WHO-recommended diseases requiring palliative care.

**Materials and methods:**

This study analyzed Cause of Death Statistics and Resident Registration Population Statistics from 2014 to 2023, provided by the Ministry of Data and Statistics (MODS). Age-standardized rates (ASR) and annual percentage changes (APC) were calculated to examine mortality trends by demographic and geographic factors.

**Results:**

The total number of deaths of the WHO-recommended diseases requiring palliative care increased by 16.4%, while the trends for ASR is decreasing. In particular, as deaths at home began to increase around 2019, the previous downward trend was reversed, and a significant sex gap was observed.

**Conclusion:**

The increasing mortality burden from WHO-recommended diseases highlights the urgent need for expanded palliative care services in the Republic of Korea. While age-standardized mortality rates have generally declined, the rise in home-based deaths and continued demographic shifts reflect the importance of targeted interventions and improved access to home-care services.

## Introduction

With rapid socioeconomic development, the population is undergoing an epidemiological transition, shifting from a disease pattern dominated by infectious diseases to one centered on degenerative and chronic diseases. This shift is partly attributable to the nutritional transition characterized by increased consumption of fast food, processed sugars, and fats, which has become a major driver further increasing the disease burden of noncommunicable diseases (NCDs). Simultaneously, the dramatic advancement of medical technologies, such as antibiotics and anticancer drugs, has extended life expectancy, further altering the global disease burden (1–3). This epidemiologic shift is affecting global disease patterns and is high on the public health agenda, particularly the rise of chronic and degenerative diseases, which can require lifelong care and lead to excessive healthcare spending (4, 5).

In 2021, seven out of 10 of the leading causes of death worldwide were non-communicable diseases, accounting for 38% of all deaths. For cancer, including non-melanoma skin cancers, the total number of deaths increased from 8.8 million in 2015 to 9.8 million in 2021. Among the leading cancers, trachea, bronchus, and lung cancers accounted for 19% at 1.85 million. For diabetes, the total number of deaths increased from 1.31 million in 2015 to 1.61 million in 2021. For Cardiovascular diseases (CVDs), the total number of deaths increased from 17.5 million in 2015 to 19.2 million in 2021, with ischemic heart disease, a type of CVDs, ranking as the number one cause of death (6–10).

To effectively manage the burden and suffering caused by serious illnesses, the World Health Organization (WHO) emphasizes the need to introduce palliative care, which aims to alleviate pain and improve patients’ quality of life. The WHO projects that serious health-related suffering amenable to such interventions will increase by 87% by 2060. Conditions requiring palliative care include various life-threatening chronic diseases such as cancer, human immunodeficiency virus (HIV) infection and acquired immunodeficiency syndrome (AIDS), cardiovascular disease (CVD), neurodegenerative diseases, and chronic respiratory diseases. This scope aligns with the concept of hospice defined in the 「Act on Hospice and Palliative Care and Decisions on Life-Sustaining Treatment for Patients at the End of Life」, which explicitly includes the diseases recommended by the WHO (11, 12). Palliative care is vital for alleviating patient suffering, improving quality of life, and reducing unnecessary healthcare expenditures. However, while approximately 56.8 million people worldwide require palliative care each year, only about 14% of the global population has access to the highest standard of palliative care (11, 13, 14).

Globally, cancer deaths rose from 4.8 million in 1990 to 9.8 million in 2021, but the age-standardized mortality rate decreased from 153.5 to 116.5 per 100,000 population. Similarly, while CVD deaths rose from 12.1 million in 1990 to 18.6 million in 2019, the age-standardized mortality rate declined significantly. These findings suggest that improvements in age-standardized mortality rates did not directly translate into reductions in absolute death counts, and that cancer and CVD remain major global causes of death (15, 16). This persistent mortality burden emphasizes a widening gap between the demand for and supply of palliative care services globally.

In 2021, more than half of the top 10 causes of death in the Republic of Korea were diseases for which the WHO has emphasized the need for palliative care. The population structure is changing rapidly due to low birth rates and aging, with those aged 65 and over expected to account for 46.4% of the population by 2070. This aging trend is increasing healthcare costs, particularly for chronic diseases such as dementia. Cancer incidence has increased by 36.4% in 2021 compared to 1999 and deaths from CVDs rose by 12.4% per 100,000 population from 2009 to 2018, with heart failure deaths surging by 96.0% (17–23). Expanding palliative care in chronic disease management is crucial globally and in the Republic of Korea. However, there is limited research on the trends of conditions requiring palliative care in the Republic of Korea. To address this gap, we analyze trends in conditions identified by the WHO as requiring palliative care, using cause-of-death statistics and resident registration population Statistics from the Ministry of Data and Statistics (MODS) from 2014 to 2023.

## Materials and methods

### Data source

This trend analysis utilized cause-of-death statistics published annually by MODS and resident registration population Statistics (January 1, 2014 to December 31, 2023). Cause-of-death statistics include sociodemographic information on individuals who died in the Republic of Korea. Under the 「Act on the Registration of Family Relations」, death reports must be filed with the competent administrative agency, accompanied by a death certificate or post-mortem examination report issued by a physician. To ensure data accuracy and reliability, MODS applies a multistage quality control process. First, submitted records undergo automated logical checks within the Vital Statistics System to identify inconsistencies. Second, records are cross-verified by linking with administrative data from external agencies to supplement missing or unclear information. Finally, trained coders assign the underlying cause of death based on the Korean Standard Classification of Diseases and Causes of Death (KCD), which is based on the International Classification of Diseases, 10th Revision (ICD-10), following the WHO guidelines for selecting and revising causes of death (24). This study focuses specifically on the resident population data by first generation by administrative region. The data is open data, and no ethical approval was required to use the data.

### Data collection

Out of all causes of death, the number of deaths targeted diseases that require palliative care as published by the WHO: Cancer (C00-C97); AIDS (B20-B24); CVDs (I00-I99); Chronic liver diseases (K70-K77); Kidney failure (N17-N19); Chronic respiratory failure (J44-J46); Diabetes (E10-E14); Multiple sclerosis (G35); Parkinson’s disease (G20); Dementia (F00-F03, G30, G311); Rheumatoid arthritis (M05, M06); Drug-resistant tuberculosis (U84. 30, U84.31, U88.2); congenital abnormalities (Q00-Q99); other (other codes). Sociodemographic variables of the deceased were categorized as follows: Age (20–29, 30–39, 40–49, 50–59, 60–69, 70 + years); Sex (male, female); Place of residence (Capital and Metropolitan, Rural); Place of death (home, medical institutions, others). Individuals younger than 20 years were excluded from the analysis, and deaths with unknown age information were omitted. The “others” category for the place of deaths includes social welfare facilities, public facilities, roads, and service facilities.

### Statistical analysis

To understand the general characteristics of overall mortality from WHO-recommended diseases for palliative care, the data were presented in terms of frequency (N) and percentage (%). Trends in the number of deaths by year, both total and by sex, were also analyzed and visualized. The Age-standardized mortality rates (ASR per 100,000 population) were calculated using the direct standardization method to control for changes in the population age structure over time. The standard population used for adjustment was the 2014 resident registration mid-year population of the Republic of Korea, which corresponds to the baseline year of this study. The population aged 20 years and older was stratified into six age groups (20–29, 30–39, 40–49, 50–59, 60–69, and ≥70 years), and age-specific mortality rates were weighted according to the age distribution of the 2014 standard population. This approach allows for a consistent comparison of mortality trends across the 10-year study period (2014–2023).

Subsequently, an annual percent change (APC) analysis was conducted to examine temporal trends in the ASR of WHO-recommended diseases for palliative care. In addition, to assess whether mortality trends differed before and after the COVID-19 period, we estimated APCs separately for the pre-COVID-19 (2014–2019) and post-COVID-19 (2020–2023) periods using a log-linear regression model. APC was calculated as 100 × (e^*β*^ − 1). Differences in APCs between the two periods were evaluated using a model that included year, period, and an interaction term between year and period. This analysis utilized the Joinpoint Regression Program (Joinpoint Trend Analysis Software, Version 5.3.0; Surveillance Research Program, National Cancer Institute, Bethesda, MD, United States) to identify trend changes at joinpoints and estimate segment-specific APC. A log-linear joinpoint regression model was applied to the natural logarithm of the ASR, assuming continuous linear trends between joinpoints. The final model was selected based on the weighted Bayesian information criterion. Constant variance (homoscedasticity) and uncorrelated errors were specified between annual observations, and 95% confidence intervals (CI) for APC were calculated using the program’s parametric method (25). Additionally, APC analyses were performed for annual death frequency (N) and rate (%) by place of death for each disease. Data were analyzed using SAS software version 9.4 (SAS Institute, Cary, NC, United States), and *p* < 0.05 were considered statistically significant.

## Results

### Demographic and geographic characteristics of deaths

Among the diseases with the high number of deaths, cancer was the leading cause among males, accounting for (*n* = 495,745; 61.8%) of male deaths. In contrast, dementia predominantly affected females, with (*n* = 73,344; 69.2%) of dementia deaths occurring among females. Geographically, deaths from chronic respiratory failure and dementia were more prevalent in rural areas, at (*n* = 37,665; 66.8%) and (*n* = 65,065; 61.4%) respectively. Conversely, AIDS resulted in more deaths (*n* = 445; 56.9%) in capital and metropolitan areas. In terms of age, most deaths including (*n* = 102,580; 96.7%) of deaths from dementia—were concentrated among those aged 70 and older. AIDS presented a different pattern, with (*n* = 76; 9.7%) of deaths occurring in the 30–39 age group (Table 1).

**Table 1:** Sociodemographic characteristics of deaths from WHO-recommended diseases for palliative care in the Republic of Korea, 2014–2023.

	No. of deaths (%)												
	Cancer	Cardiovascular diseases	Diabetes	Chronic liver diseases	Chronic respiratory diseases	Dementia	Kidney failure	Parkinson’s disease	Rheumatoid arthritis	Congenital abnormalities	AIDS	Multiple sclerosis	Other^a^
Age														*N* (%)	*N* (%)	*N* (%)	*N* (%)	*N* (%)	*N* (%)	*N* (%)	*N* (%)	*N* (%)	*N* (%)	*N* (%)	*N* (%)	*N* (%)
20–29	2,702 (0.3)	1,466 (0.2)	192 (0.2)	189 (0.3)	26 (0.1)	1 (0.0)	69 (0.1)	1 (0.0)	3 (0.2)	211 (14.9)	20 (2.6)	1 (0.7)	21,290 (1.8)
30–39	9,413 (1.2)	5,129 (0.8)	539 (0.6)	2,117 (3.1)	45 (0.1)	4 (0.0)	267 (0.4)	11 (0.0)	7 (0.5)	179 (12.6)	76 (9.7)	4 (2.8)	31,682 (2.7)
40–49	35,098 (4.4)	17,556 (2.8)	2,298 (2.4)	10,237 (14.8)	193 (0.3)	50 (0.1)	1,011 (1.7)	78 (0.2)	24 (1.6)	176 (12.4)	160 (20.5)	24 (17.0)	57,501 (4.9)
50–59	100,447 (12.5)	40,943 (6.6)	7,225 (7.5)	20,482 (29.6)	1,144 (2.0)	511 (0.5)	3,410 (5.7)	733 (1.9)	67 (4.6)	246 (17.3)	234 (29.9)	37 (26.2)	97,933 (8.3)
60–69	171,229 (21.3)	67,015 (10.8)	13,433 (13.9)	15,625 (22.5)	4,306 (7.6)	2,811 (2.7)	7,191 (11.9)	3,300 (8.6)	259 (17.6)	226 (15.8)	155 (19.8)	33 (23.4)	122,866 (10.5)
≥70	483,494 (60.3)	490,330 (78.8)	72,897 (75.4)	20,649 (29.7)	50,673 (89.9)	102,580 (96.7)	48,322 (80.2)	34,162 (89.3)	1,109 (75.5)	383 (27.0)	137 (17.5)	42 (29.9)	843,483 (71.8)
Sex														*N* (%)	*N* (%)	*N* (%)	*N* (%)	*N* (%)	*N* (%)	*N* (%)	*N* (%)	*N* (%)	*N* (%)	*N* (%)	*N* (%)	*N* (%)
Male	495,745 (61.8)	295,061 (47.4)	49,033 (50.8)	51,545 (74.4)	36,835 (65.3)	32,613 (30.8)	30,180 (50.1)	17,492 (45.7)	423 (28.8)	714 (50.2)	721 (92.2)	60 (42.6)	625,514 (53.2)
Female	306,638 (38.2)	327,378 (52.6)	47,551 (49.2)	17,754 (25.6)	19,552 (34.7)	73,344 (69.2)	30,090 (49.9)	20,793 (54.3)	1,046 (71.2)	707 (49.8)	61 (7.8)	81 (57.4)	549,241 (46.8)
Region													
Capital and metropolitan	333,281 (41.5)	246,870 (39.7)	40,232 (41.7)	28,627 (41.3)	18,722 (33.2)	40,892 (38.6)	24,050 (39.9)	16,817 (43.9)	595 (40.5)	607 (42.7)	445 (56.9)	65 (46.1)	449,581 (38.3)
Rural	469,102 (58.5)	375,569 (60.3)	56,352 (58.3)	40,672 (58.7)	37,665 (66.8)	65,065 (61.4)	36,220 (60.1)	21,468 (56.1)	874 (59.5)	814 (57.3)	337 (43.1)	76 (53.9)	725,174 (61.7)

AIDS, Acquired Immune Deficiency Syndrome; WHO, World Health Organization. Percentages represent the proportion of total deaths within each category. Data on drug-resistant tuberculosis were excluded as no deaths were reported during the period. ^a^includes all diseases not classified under WHO-recommended diseases for palliative care. AIDS, acquired immune deficiency syndrome; WHO, World Health Organization.

### Ten-year trends of increasing deaths from WHO-recommended diseases for palliative care

**Figure 1:**
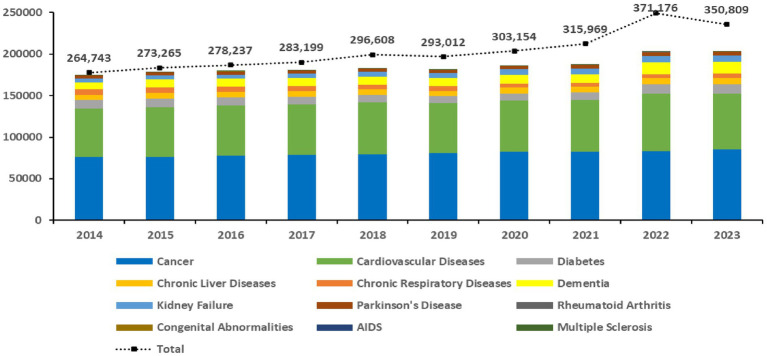
Ten-year trends in the number of deaths from WHO-recommended diseases for palliative care in the Republic of Korea. This stacked bars represent the total number of deaths for each specific disease category, while the dashed line indicates the total number of deaths from all causes. Data on drug-resistant tuberculosis were excluded as no deaths were reported during the period. AIDS, acquired immune deficiency syndrome; WHO, World Health Organization.

From 2014 to 2023, the total deaths from WHO-recommended diseases for palliative care in the Republic of Korea steadily increased, with the exception of a slight decrease in 2019. The total deaths rose by 16.4%, from 174,435 in 2014 to 203,088 in 2023. This increase was primarily driven by major diseases such as cancer, CVDs, and dementia, which accounted for more than 80.0% of all deaths. Deaths from cancer increased from 76,355 in 2014 to 85,116 in 2023, an increase of 10.9%. Deaths from CVDs rose from 57,705 in 2014 to 67,490 in 2023, an increase of 17.0%. Deaths from dementia showed the most significant increase, jumping by 65.9%, from 8,587 in 2014 to 14,247 in 2023 (Figure 1).

### Ten-year decline in age-standardized rates for WHO-recommended diseases for palliative care

The ASR per 100,000 population for WHO-recommended diseases for palliative care in the Republic of Korea generally showed declining trends. The ASR for cancer, which had the greatest impact, decreased significantly from 187.4 in 2014 to 151.9 in 2023, representing a 18.9% reduction. Similarly, CVDs decreased from 141.6 in 2014 to 119.2 in 2023, marking a 15.8% reduction. However, dementia showed the opposite trends, with the ASR increasing from 21.1 in 2014 to 24.5 in 2023, representing a 16.1% rise (Table 2).

**Table 2:** 10-year trends in age-standardized mortality rates of WHO-recommended diseases for palliative care in the Republic of Korea, 2014–2023.

Type	Year									
	2014	2015	2016	2017	2018	2019	2020	2021	2022	2023
Cancer	187.4	181.6	178.4	172.7	166.9	164.8	160.7	157.2	153.3	151.9
Cardiovascular diseases	141.6	141.2	138.1	133.9	132.1	121.4	120.8	118.4	126.2	119.2
Diabetes	25.8	25.0	22.4	20.1	18.4	16.3	16.4	16.9	20.3	19.5
Chronic liver diseases	16.3	16.4	15.8	15.5	15.2	14.1	14.8	14.8	15.3	14.5
Chronic respiratory diseases	15.7	15.9	14.1	12.9	12.0	10.7	9.3	8.5	9.3	9.2
Dementia	21.1	22.5	21.0	20.2	20.2	20.6	20.3	19.3	25.3	24.5
Kidney failure	11.0	11.9	11.9	11.5	11.8	12.1	12.7	13.1	13.5	13.2
Parkinson’s disease	8.2	8.1	8.2	8.1	8.2	6.9	6.8	7.2	8.5	7.9
Rheumatoid arthritis	0.4	0.3	0.3	0.3	0.2	0.3	0.2	0.3	0.3	0.3
Congenital abnormalities	0.3	0.3	0.3	0.3	0.3	0.3	0.4	0.3	0.3	0.4
AIDS	0.3	0.2	0.2	0.2	0.2	0.2	0.1	0.1	0.1	0.1
Multiple sclerosis	0.1	0.0	0.0	0.0	0.0	0.0	0.0	0.0	0.0	0.0
Other^a^	221.6	226.2	228.3	227.9	242.8	230.1	233.8	250.5	313.5	268.1

Values represent age-standardized rates per 100,000 population. Data on drug-resistant tuberculosis were excluded as no deaths were reported during the period. ^a^includes all diseases not classified under WHO-recommended diseases for palliative care. AIDS, acquired immune deficiency syndrome; WHO, World Health Organization.

### Changes in mortality trends before and after the COVID-19 period

The trend in age-standardized mortality rates by place of death differed between the pre-COVID-19 period (2014–2019) and the post-COVID-19 period (2020–2023). For all deaths, the trend was decreasing before COVID-19, with −1.5 (APC, 95% CI: −3.3, 0.2), but shifted to an increasing trend afterward, with 2.9 (APC, 95% CI: −0.5, 6.3); however, the difference in APC between the two periods was not statistically significant. Conversely, deaths occurring at home showed a decreasing trend before COVID-19, with −4.6 (APC, 95% CI: −6.7, −2.4), but shifted to an increasing trend afterward, with 2.7 (APC, 95% CI: −1.5, 7.2), with the change in trend between the two periods being statistically significant (Table 3).

**Table 3:** Changes in annual percentage changes of age-standardized mortality rates (ASR) per 100,000 population for deaths for WHO-recommended diseases for palliative care before and after COVID-19 by place of death in the Republic of Korea, 2014–2023.

WHO-recommended diseases for palliative care	Pre-COVID 19 (2014–2019)	Post-COVID 19 (2020–2023)	*P*-value
ASR for all death	−1.5	2.9	0.062
ASR for home deaths	−4.6	2.7	0.024
ASR for medical institution deaths	−0.7	2.7	0.118
ASR for other place deaths^a^	−3.0	4.4	0.016

APCs were estimated separately for the pre-COVID-19 (2014–2019) and post-COVID-19 (2020–2023) periods using a log-linear regression model of ASR on calendar year [ln(ASR) = α + β·year], and APC was calculated as 100 × (e^β − 1). ASRs were calculated per 100,000 population and age-standardized to the 2014 resident registration population of the Republic of Korea (aged ≥20 years). *p*-values test whether APCs differed between the two periods based on the interaction between calendar year and period. ^a^The “other place” category includes social welfare facilities, public facilities, roads, and service facilities. APC, annual percentage change; ASR, age-standardized mortality rate; WHO, World Health Organization.

### Ten-year shift in annual percentage changes in WHO-recommended diseases for palliative care

**Figure 2:**
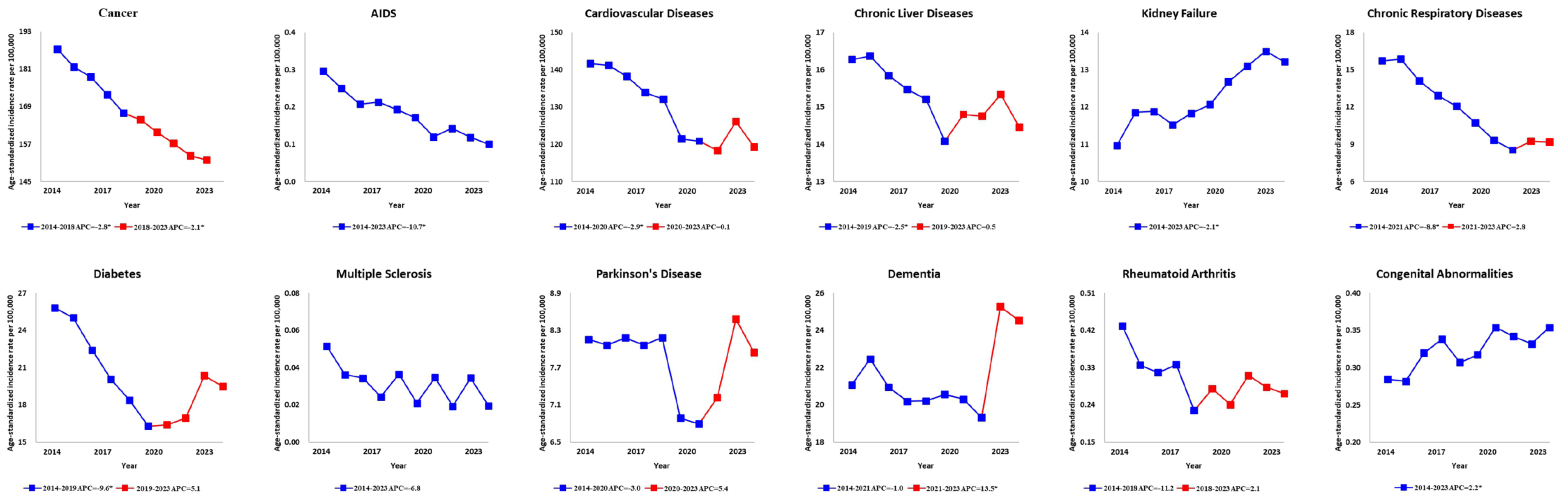
Ten-year trends in annual percentage changes of age-standardized mortality rates per 100,000 population for WHO-recommended diseases for palliative care in the Republic of Korea. Figure shows the trends for the total population. The solid lines represent the trends in age-standardized mortality rates per 100,000 population, standardized to the 2014 resident registration population of the Republic of Korea (aged ≥20 years). Red points indicate joinpoints, reflecting a significant change in the trend. Asterisks (*) indicate statistical significance (*p* < 0.05). Data on drug-resistant tuberculosis were excluded as no deaths were reported during the period. AIDS, acquired immune deficiency syndrome; APC, annual percentage change; WHO, World Health Organization.

Before 2019, many of the declining trends for most WHO-recommended diseases for palliative care experienced a turning point around 2020, resulting in changes in these trends. For instance, cancer showed a continuous decline of −2.8 (APC 95% CI: −3.3, −2.2) from 2014 to 2018 and −2.1 (APC, 95% CI: −2.5, −1.7) from 2018 to 2023. In contrast, diabetes saw a decrease from 2014 to 2019, with −9.6 (APC, 95% CI: −12.9, −6.2), but this trend reversed slightly after 2019, increasing with 5.1 (APC, 95% CI: −0.3, 10.8) (Figure 2).

### Place of deaths and changing patterns in annual percentage changes in WHO-recommended diseases for palliative care

Before 2019, most WHO-recommended diseases for palliative care showed declining trends in deaths occurring at home, but this trend reversed around 2019. For cancer, there was a decrease from 2014 to 2018 with −8.7 (APC, 95% CI: −22.4, 7.4), but the trends reversed from 2018 to 2023, increasing with 9.1 (APC, 95% CI: −2.8, 22.4). Similarly, CVDs showed a decrease from 2014 to 2019 with −4.3 (APC, 95% CI: −7.6, −0.9), but the trends reversed between 2019 and 2023, increasing with 4.7 (APC, 95% CI: −0.3, 10.1). In contrast, the trends for deaths occurring in medical institutions generally continued to decrease or saw only slight increases. For cancer, there was a continuous decline from 2014 to 2023 with −2.6 (APC, 95% CI: −3.0, −2.2). Similarly, the trend for deaths from CVDs occurring in medical institutions decreased by −2.3 (APC, 95% CI: −2.9, −1.6) from 2014 to 2023. Diabetes also showed one of the most significant trends changes. In other areas, diabetes showed a decline from 2014 to 2019 with −13.1 (APC, 95% CI: −15.8, −10.2), but this trend dramatically reversed between 2019 and 2023, increasing with 11.3 (APC, 95% CI: 6.4, 16.5).

**Figure 3:**
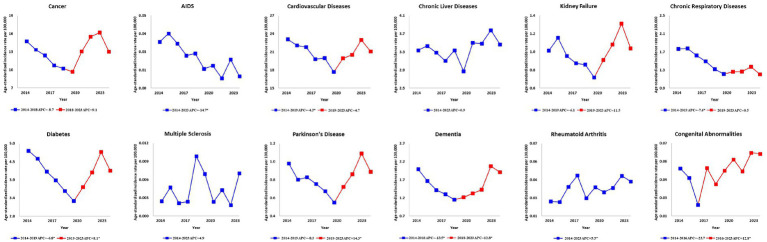
Ten-year trends in annual percentage changes of age-standardized mortality rates per 100,000 population for deaths occurring at home from WHO-recommended diseases for palliative care in the Republic of Korea. Figure shows the trends for the total population. The solid lines represent the trends in age-standardized mortality rates per 100,000 population for deaths occurring at home, standardized to the 2014 resident registration population of the Republic of Korea (aged >20 years). Red points indicate joinpoints, reflecting a significant change in the trend. Asterisks (*) indicate statistical significance (*p* < 0.05). Data on drug-resistant tuberculosis were excluded as no deaths were reported during the period. AIDS, acquired immune deficiency syndrome; APC, annual percentage change; WHO, World Health Organization.

When examining trends by disease proportion, similar changes in the place of death were observed, indicating that the overall trends have become more pronounced (Figure 3; Supplementary Figures 1, 2).

### Sex disparity

Cancer, the leading cause of death among males, increased by 9.2%, from 47,722 deaths in 2014 to 52,091 in 2023. Meanwhile, CVDs were the leading cause of death among females, increasing by 15.6%, from 30,259 in 2014 to 34,972 in 2023. Significant sex differences were also observed in certain diseases, with AIDS mortality 11 times higher among males than females. In terms of ASR, chronic liver disease showed contrasting patterns between males and females. Among males, the ASR for chronic liver disease decreased by 17.0%, from 25.0 in 2014 to 20.7 in 2023. However, among females, it increased by 5.1%, from 7.7 in 2014 to 8.1 in 2023. When examining the APC for Parkinson’s disease. In males, the APC decreased slightly from 2014 to 2020, to −3.5 (APC, 95% CI: −7.3, 0.4), but then increased sharply after 2020, reaching 6.2 (APC, 95% CI: −5.6, 19.4). In contrast, females remained relatively stable at −0.6 (APC, 95% CI: −2.6, 1.4) (Supplementary Tables 1, 2; Supplementary Figures 3, 4).

## Discussion

The study analyzed mortality trends of WHO-recommended diseases for palliative care in the Republic of Korea from 2014 to 2023 and found that the total number of deaths increased by 16.4%. The main causes were cancer, CVDs and dementia, which together accounted for more than 80.0% of the deaths. Our study highlights the potential impact of the COVID-19 pandemic on shifts in the place of end-of-life care. The age-standardized mortality rate (ASR) for deaths at home showed a notable increase of approximately 34%, rising from 84.2 per 100,000 population in 2019 to 113.0 in 2022. In contrast, the ASR for deaths in medical institutions temporarily declined from 457.8 in 2019 to 447.3 in 2020, before rebounding to 508.7 in 2022. This increase in home deaths may be associated with strict infection control measures, such as visitation bans, which likely influenced the quality of end-of-life care experiences in hospitals. Furthermore, previously reported disruptions including the conversion of hospital beds for COVID-19 use and reduced healthcare utilization by non-COVID patients suggest that these shifts were related to the redistribution of medical resources and limited healthcare access (26–28).

Significant sex disparities were observed in mortality trends. Males generally exhibited higher mortality rates, particularly for cancer, which may be attributed to a higher prevalence of traditional risk factors, such as smoking, and delayed health-seeking behaviors (29, 30). However, a contrasting pattern was identified regarding Chronic Liver Diseases While the ASR for Chronic Liver Diseases in males declined from 20.8 in 2019 to 20.7 in 2023, the rate in females showed an upward trend, rising from 7.4 in 2019 to 8.1 in 2023. This pattern aligns with recent national data indicating a rise in high-risk drinking and monthly binge drinking rates among women (31). These findings suggest that evolving gender-specific lifestyle patterns, particularly increased alcohol consumption in females, may be influencing mortality trends for alcohol-related conditions.

These findings suggest a growing need for palliative care services and highlight the urgency to expand and adapt palliative care to meet future demands.

Comparing these trends with those observed in the United States (US), the United Kingdom (UK), and Taiwan provides further insight. In both the US and the UK, the number of cancer deaths has increased in recent years, but ASR have decreased. In the US, cancer deaths increased by 4.9% in 2022 compared to 2013, while ASR per 100,000 population decreased by 13.4%. Similarly, the UK saw a 5.7% increase in cancer deaths between 2011 and 2013 and 2019–2021, but a 7.8% decrease in ASR per 100,000 population (32–35).

In the US, it is estimated that by 2030, 80% of people aged 65 and older will have at least one chronic condition. Palliative care, including community-based services, can enhance quality of life and symptom management. Palliative care began in 1974 and was institutionalized in 1982 under the Medicare Hospice Benefit, which introduced a prospective payment system that incentivized its growth. Initially focused on terminal cancer patients, palliative care has expanded to include those with chronic illnesses like AIDS and heart failure. Currently, over 3,600 organizations provide palliative care annually, serving more than 1 million patients (36–38).

In the UK, an estimated 75% of people in England and Wales will benefit from palliative care as they near the end of their lives, with demand expected to increase by 25% by 2040. Palliative care began at St. Christopher’s Hospice in 1967, and the National Health Service (NHS) has expanded services since the late 1990s. The 2008 End of Life Care Strategy aimed to provide free palliative care for all patients. Charities like Marie Curie collaborate with the NHS to offer home-based care, improving access. Various palliative care services now operate across the UK, including community-based services linked to hospitals, day care centers, and home care. As of 2001, there were 53 specialist units and over 69 home care teams (39–41).

Similar trends were observed in Taiwan, where cancer deaths increased by 15.3% in 2021 compared to 2013, while the ASR per 100,000 population decreased by 9.1% (42, 43).

In Taiwan, over 40,000 cancer patients die annually, driving increased demand for palliative care. Palliative care services began at Mackay Memorial Hospital in 1990, and by 1995, the National Health Insurance covered palliative care for terminal cancer patients. In 2000, Taiwan became the first country in Asia to establish a legal framework for palliative care. The services have since expanded to include chronic conditions like dementia and heart failure. In 2009, the Palliative Care Consultation Service was introduced to cover a broader range of patients. As of 2015, there were 77 home care programs and 51 inpatient institutions providing comprehensive palliative care (44–46).

Additionally, when examining changes in the place of death in the US, there was a slight shift in Washington State, where Medicaid-enrolled cancer patients were unable to receive palliative care and died at home during the COVID-19 pandemic (20% before the pandemic, 38% during the pandemic) (47).

Similar to the findings of the study’s trends analysis, the US, UK, and Taiwan also showed an increase in the number of deaths from cancer, one of the WHO-recommended diseases for palliative care, while ASR decreased. This suggests that public health interventions and medical advances are having an impact. However, as the number of deaths continues to rise due to an aging population, palliative care services will need to expand to cover more conditions. Early palliative care (EPC) has been shown to improve quality of life by 0.14 [SMD (Standardized Mean Difference), 95% CI: 0.62, 0.223] and reduce symptom burden by 0.14 (SMD, 95% CI: 0.01, 0.26) in advanced cancer patients, underscoring the benefits of earlier intervention. Currently, palliative care services are primarily provided to cancer patients, but it is important to reduce the burden of disease by providing early access to patients with a variety of conditions, including chronic diseases. The provision of palliative care services for major diseases with a high burden of disease can contribute to improving the quality of life of patients, reducing societal costs, and increasing the efficiency of healthcare systems. To effectively enable this, a variety of policy approaches are needed, including legislation, national guidelines, financial support, health system integration, community networking, and training of professionals (48–52).

### Strengths and limitations

This study has several strengths. First, the cause-of-death statistics data, regularly updated by MODS, covers the entire population of the Republic of Korea, enabling a comprehensive assessment of the relationship between medical practices and policies. Second, using ASR and APC allows for comparing long-term mortality trends while accounting for demographic changes. Despite these strengths, several limitations exist. First, cause-of-death statistics capture only underlying causes of death, excluding healthcare utilization information particularly hospital electronic medical records which may lead to misclassification. Second, incomplete ICD-10 codes might have partially led to in misclassification or underestimation. Third, we were not able to incorporate all of the individual-level sociodemographic factors related to place of death, such as availability of a caregiver toward the end-of-life.

## Conclusion

This study provides a comprehensive analysis of 10-year mortality trends for WHO-recommended diseases requiring palliative care in the Republic of Korea. The findings demonstrate the growing burden of the diseases recommended for palliative care by the WHO, especially for cancer, cardiovascular diseases, and dementia in an aging population, along with an urgent need to expand palliative care services. While decreases in age-standardized mortality rates suggest improvements in medical care and early detection, the rising number of dementia-related deaths, age and sex disparities highlight the need for targeted health system interventions. Shifts in the place of death reflect changes in preferences for end-of-life care and increased demand for home-based care.

## Data Availability

The original contributions presented in the study are included in the article/Supplementary material, further inquiries can be directed to the corresponding author.
