# Correction: Trends in mortality of the WHO-recommended diseases for palliative care in the Republic of Korea, 2014–2023

**DOI:** 10.3389/fpubh.2026.1813585

**Published:** 2026-03-19

**Authors:** Minju Kim, Kyuwoong Kim, Woorim Kim, Eun Jeong Nam, Su Yeon Kye, Jin Young Choi

**Affiliations:** 1 National Cancer Control Institute, National Cancer Center, Goyang, Republic of Korea; 2 Graduate School of Cancer Science and Policy, National Cancer Center, Goyang, Republic of Korea

**Keywords:** cause of death, mortality, palliative care, Republic of Korea, World Health Organization

References 17, 21, and 24 were erroneously written.

Reference 17 was erroneously written as “Korea National Statistical Office. *Population projections for Korea (2020*~*2070)*. Daejeon: Statistics Korea (2021)”. The correct reference is “Statistics MoDa. *Population projections for Korea (2020–2070)*. Daejeon, Republic of Korea: Ministry of Data and Statistics (2021)”.

Reference 21 was erroneously written as “Korea National Statistical Office. *Causes of death statistics in 2021*. Daejeon: Statistics Korea (2022)”. The correct reference is “Statistics MoDa. *Causes of Death Statistics in 2021*. Daejeon, Republic of Korea: Ministry of Data and Statistics (2022)”. Reference 24 was erroneously written as “Korea National Statistical Office. *Quality report on cause of death statistics*. Daejeon, Republic of Korea: Ministry of Data and Statistics. (2022)”. The correct reference is: “Statistics MoDa. *Quality Report on Cause of Death Statistics*. Daejeon, Republic of Korea: Ministry of Data and Statistics (2022)”.

In the Materials and methods section in the **Abstract**, the acronym of the data provider ministry was incorrectly written as “(MDAS)”. The correct acronym is “(MODS)”.

In the Results section in the **Abstract**, there was a grammatical error in the sentence:

“The total number of deaths of the due to WHO-recommended diseases requiring palliative care increased by 16.4%, while the trends for ASR is decreasing.”

The corrected sentence is:

“The total number of deaths of the WHO-recommended diseases requiring palliative care increased by 16.4%, while the trends for ASR is decreasing.”

The equation in **Materials and methods**, *Statistical analysis*, was erroneously given as:

“APC was calculated as 100 × (e^^^β – 1).”

The correct equation is:

“APC was calculated as 100 × (e^β^ – 1).”

The sentence from the **Introduction**, paragraph 4, was incorrectly written as:

“This persistent mortality burden underscores a widening gap between the demand for and supply of palliative care services globally.”

This has been corrected to:

“This persistent mortality burden emphasizes a widening gap between the demand for and supply of palliative care services globally.”

The wording in the **Introduction**, paragraph 5, was incorrect, and the data source was inaccurately written as:

“To address this gap, we analyze trends in conditions identified by the WHO as requiring palliative care, using cause of death and population data from Statistics Korea from 2014 to 2023.” This has been corrected to:

“To address this gap, we analyze trends in conditions identified by the WHO as requiring palliative care, using cause-of-death statistics and resident registration population Statistics from the Ministry of Data and Statistics (MODS) from 2014 to 2023.”

In the **Materials and methods** section, the data source and responsible institution were incorrectly stated as “Statistics Korea” in two instances.

The sentence in **Materials and methods**, *Data source*, previously stated:

“This trend analysis utilized cause-of-death statistics published annually by Statistics Korea and resident registration population data (January 1, 2014 to December 31, 2023).”

The corrected sentence reads:

“This trend analysis utilized cause-of-death statistics published annually by MODS and resident registration population Statistics (January 1, 2014 to December 31, 2023).”

The sentence in **Materials and methods**, *Data source*, previously stated:

“To ensure data accuracy and reliability, Statistics Korea applies a multi-stage quality control process.”

The corrected sentence reads:

“To ensure data accuracy and reliability, MODS applies a multi-stage quality control process.”

In the **Discussion**, *Strength and limitations*, the institution name and country name were incorrectly written as:

“This study has several strengths. First, the cause-of-death statistics data, regularly updated by Statistics Korea, covers the entire population of South Korea.”

This has been corrected to:

“This study has several strengths. First, the cause-of-death statistics data, regularly updated by MODS, covers the entire population of the Republic of Korea.”

The original version of this article has been updated.

