# Supplementary figures and images for: Trends in mortality of the WHO-recommended diseases for palliative care in the Republic of Korea, 2014–2023
Source: Front Public Health. 2026 Feb 11;14:1752495. doi: 10.3389/fpubh.2026.1752495 (PMC12932552; doi:10.3389/fpubh.2026.1752495)

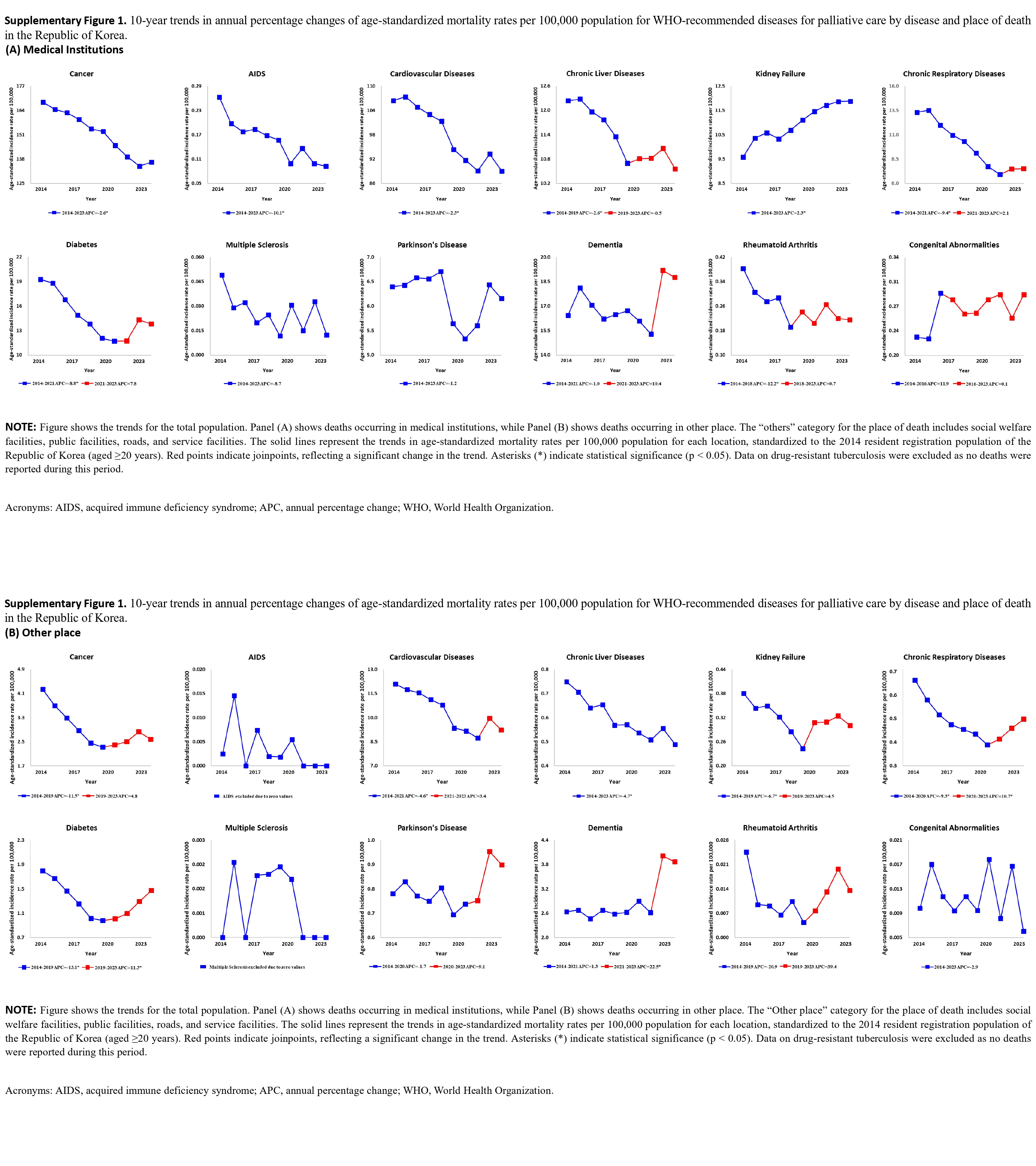

Supplement: Supplementary file 1 [file Image_1.jpeg]

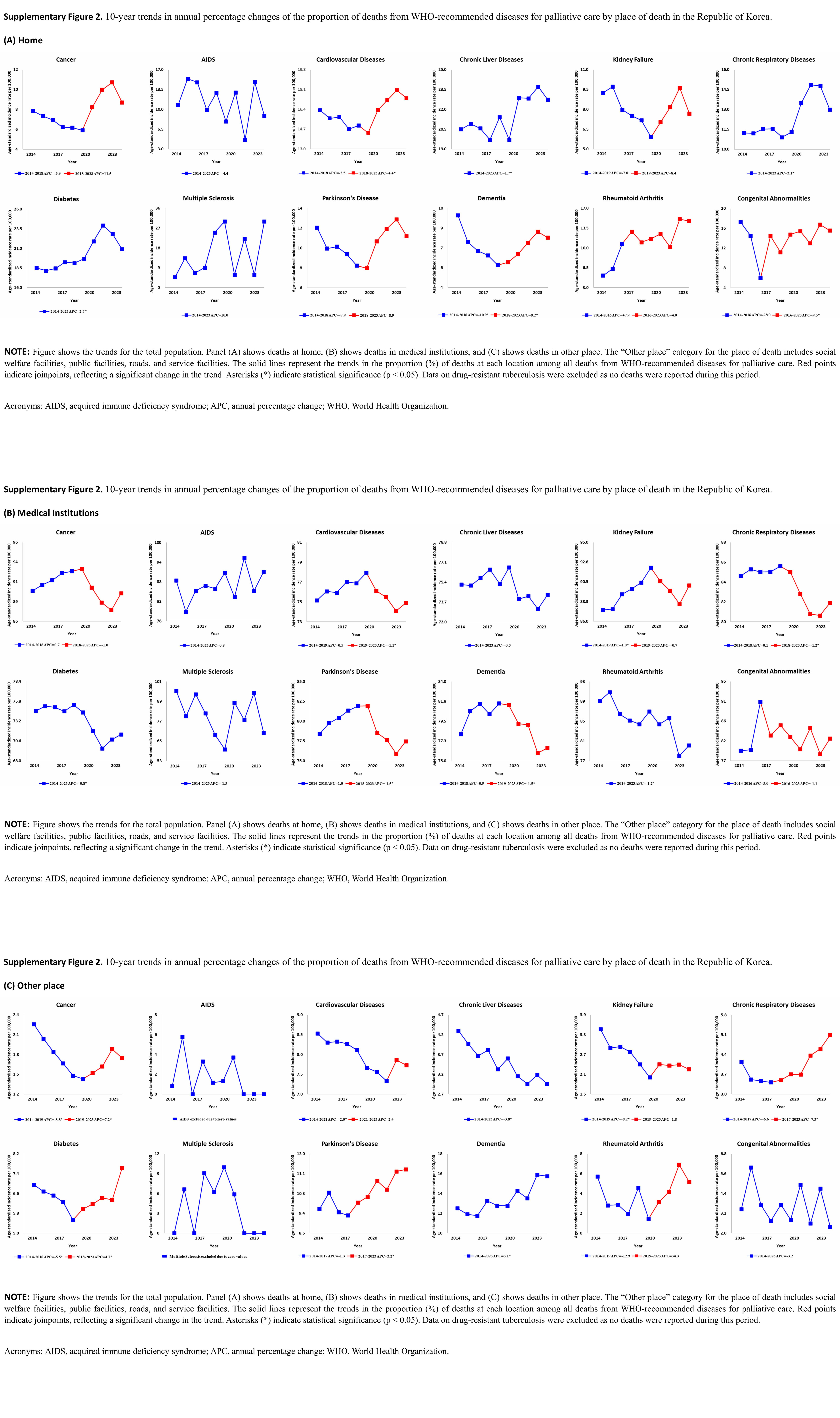

Supplement: Supplementary file 2 [file Image_2.jpeg]

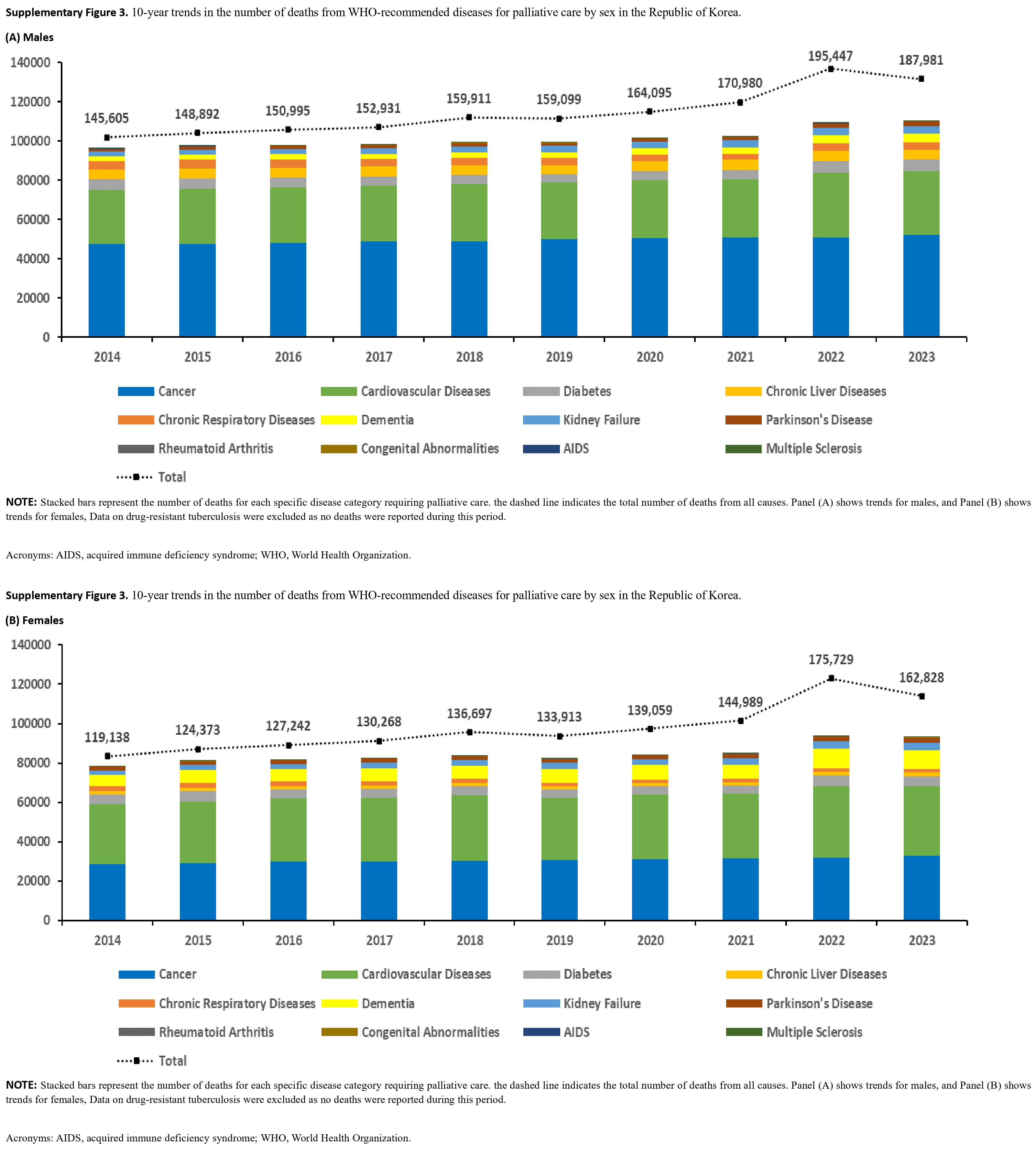

Supplement: Supplementary file 3 [file Image_3.jpeg]

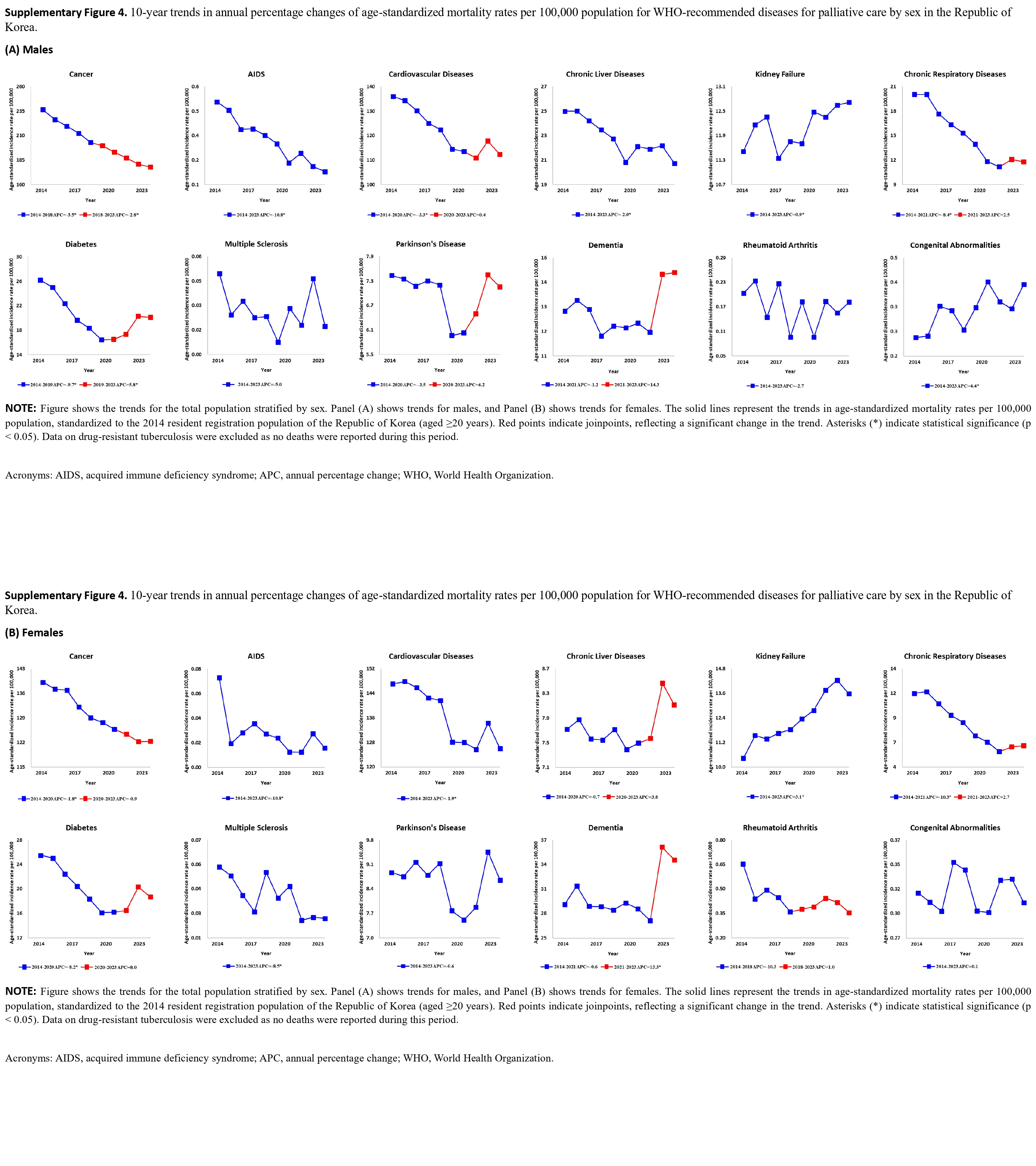

Supplement: Supplementary file 4 [file Image_4.jpeg]
