# Supplementary material for: Trends in mortality of the WHO-recommended diseases for palliative care in the Republic of Korea, 2014–2023
Source: Front Public Health. 2026 Feb 11;14:1752495. doi: 10.3389/fpubh.2026.1752495 (PMC12932552; doi:10.3389/fpubh.2026.1752495)
Supplement: Supplementary file 5 [file Table_1.docx]

**Supplementary Table 1**. 10-year trends in age-standardized mortality rates for WHO-recommended diseases for palliative care among males in the Republic of Korea.

|  | Year(%) | | | | | | | | | |
| --- | --- | --- | --- | --- | --- | --- | --- | --- | --- | --- |
| Type | 2014 | 2015 | 2016 | 2017 | 2018 | 2019 | 2020 | 2021 | 2022 | 2023 |
| Cancer | 236.5 | 226.4 | 219.6 | 212.4 | 203.1 | 199.7 | 193.1 | 187.3 | 181.0 | 177.8 |
| Cardiovascular Diseases | 136.0 | 134.3 | 130.2 | 125.1 | 122.4 | 114.5 | 113.5 | 110.8 | 117.8 | 112.3 |
| Diabetes | 26.2 | 25.0 | 22.4 | 19.7 | 18.4 | 16.4 | 16.5 | 17.3 | 20.3 | 20.1 |
| Chronic Liver Diseases | 25.0 | 25.0 | 24.2 | 23.5 | 22.7 | 20.8 | 22.1 | 21.9 | 22.2 | 20.7 |
| Chronic Respiratory Diseases | 20.0 | 20.0 | 17.7 | 16.4 | 15.3 | 14.0 | 11.8 | 11.2 | 12.1 | 11.8 |
| Dementia | 12.9 | 13.4 | 13.0 | 11.6 | 12.1 | 12.0 | 12.2 | 11.7 | 14.8 | 14.9 |
| Kidney Failure | 11.5 | 12.2 | 12.4 | 11.3 | 11.8 | 11.7 | 12.5 | 12.4 | 12.6 | 12.7 |
| Parkinson's Disease | 7.4 | 7.4 | 7.2 | 7.3 | 7.2 | 6.0 | 6.0 | 6.5 | 7.4 | 7.2 |
| Rheumatoid Arthritis | 0.2 | 0.2 | 0.1 | 0.2 | 0.1 | 0.2 | 0.1 | 0.2 | 0.2 | 0.2 |
| Congenital Abnormalities | 0.3 | 0.3 | 0.3 | 0.3 | 0.3 | 0.3 | 0.4 | 0.4 | 0.3 | 0.4 |
| AIDS | 0.5 | 0.5 | 0.4 | 0.4 | 0.4 | 0.3 | 0.2 | 0.3 | 0.2 | 0.2 |
| Multiple Sclerosis | 0.0 | 0.0 | 0.0 | 0.0 | 0.0 | 0.0 | 0.0 | 0.0 | 0.0 | 0.0 |
| Other | 245.1 | 247.0 | 246.9 | 244.0 | 259.5 | 246.6 | 247.7 | 263.6 | 316.2 | 278.0 |

NOTE: Values represent age-standardized mortality rates per 100,000 population, standardized to the 2014 resident registration population of the Republic of Korea (aged ≥20 years). Data on drug-resistant tuberculosis were excluded as no deaths were reported during the period.

Acronyms: AIDS, acquired immune deficiency syndrome; WHO, World Health Organization.

**Supplementary Table 2**. 10-year trends in age-standardized mortality rates (ASRs) for WHO-recommended diseases for palliative care among females in the Republic of Korea.

|  | Year(%) | | | | | | | | | |
| --- | --- | --- | --- | --- | --- | --- | --- | --- | --- | --- |
| Type | 2014 | 2015 | 2016 | 2017 | 2018 | 2019 | 2020 | 2021 | 2022 | 2023 |
| Cancer | 139.2 | 137.2 | 136.9 | 132.1 | 129.0 | 127.7 | 125.8 | 124.3 | 122.3 | 122.4 |
| Cardiovascular Diseases | 147.1 | 147.9 | 145.9 | 142.6 | 141.7 | 128.1 | 128.0 | 125.7 | 134.3 | 126.0 |
| Diabetes | 25.5 | 25.0 | 22.4 | 20.4 | 18.4 | 16.1 | 16.2 | 16.5 | 20.3 | 18.7 |
| Chronic Liver Diseases | 7.7 | 7.9 | 7.6 | 7.5 | 7.7 | 7.4 | 7.5 | 7.6 | 8.5 | 8.1 |
| Chronic Respiratory Diseases | 11.5 | 11.7 | 10.4 | 9.3 | 8.6 | 7.2 | 6.6 | 5.6 | 6.1 | 6.2 |
| Dementia | 29.1 | 31.4 | 28.9 | 28.8 | 28.4 | 29.3 | 28.6 | 27.1 | 36.1 | 34.5 |
| Kidney Failure | 10.4 | 11.6 | 11.4 | 11.7 | 11.8 | 12.3 | 12.8 | 13.7 | 14.2 | 13.6 |
| Parkinson's Disease | 8.9 | 8.8 | 9.2 | 8.8 | 9.1 | 7.8 | 7.5 | 7.9 | 9.5 | 8.6 |
| Rheumatoid Arthritis | 0.7 | 0.4 | 0.5 | 0.4 | 0.4 | 0.4 | 0.4 | 0.4 | 0.4 | 0.4 |
| Congenital Abnormalities | 0.3 | 0.3 | 0.3 | 0.3 | 0.3 | 0.3 | 0.3 | 0.3 | 0.3 | 0.3 |
| AIDS | 0.1 | 0.0 | 0.0 | 0.0 | 0.0 | 0.0 | 0.0 | 0.0 | 0.0 | 0.0 |
| Multiple Sclerosis | 0.1 | 0.0 | 0.0 | 0.0 | 0.1 | 0.0 | 0.0 | 0.0 | 0.0 | 0.0 |
| Other | 198.7 | 205.7 | 209.7 | 211.4 | 225.4 | 212.7 | 218.7 | 235.9 | 308.8 | 256.0 |

NOTE: Values represent age-standardized mortality rates per 100,000 population, standardized to the 2014 resident registration population of the Republic of Korea (aged ≥20 years). Data on drug-resistant tuberculosis were excluded as no deaths were reported during the period.

Acronyms: AIDS, acquired immune deficiency syndrome; WHO, World Health Organization.
